# Supplementary material for: Learning curve and surgical outcome of robotic assisted colorectal surgery with ERAS program
Source: Sci Rep. 2022 Nov 29;12:20566. doi: 10.1038/s41598-022-24665-w (PMC9709162; doi:10.1038/s41598-022-24665-w)
Supplement: Supplementary file 1 — Supplementary Information 1. [file 41598_2022_24665_MOESM1_ESM.zip › LSC Raw data-Ñ[▒K/149/P_ñw╝╨░O▒Kñσ.pdf]

[redacted]

Pathologic diagnosis:
Colon, terminal ileum, and appendix, lapa-right hemicolectomy ---a. Chronic ulcer with luminal narrowing of colon. See comment. Reactive hyperplasia of mesenteric and mesocolic lymph nodes (0/37). b. Fecalith in appendix. c. Pseudomelanosis coli. d. Diverticula.

Ancillary study for diagnosis:
IHC stain CMV (-) done on section N.

Gross description:
The specimen consists of a segment of colon, 20 cm in length, and 7 cm in proximal circumference, 5 cm in distal cut end circumference, and a segment of terminal ileum, 7 cm in length, and 4 cm in cut end circumference. An unremarkable appendix, 6x0.7x0.5 cm, is noted with fecalith. The colon mucosa show geographic ulcerative area with discontinuous lesions. There are two narrowing part with circumferental circumference of 4 cm found. The proximal one is 4 cm to ileocecal valve and the distal one is 5.5 cm to the distal cut end. A diverticulum, 1x0.5x0.5 cm, with feces found just proximal to the distal narrow part. The ileocecal vlave is deformed.
Representative part for section: A)distal cut end B)proximal cut end C)appendix D)ileocecal valve E-J)mesocollic lymph nodes (F is the other half of E; His the other half of G) K-L)distal stricture lesion (ink the narrow part) M)diverticulum V-W)mesocolic lymph nodes N-Q)proximal narrow part (in the order of prixmal part to distal end with narrow part ink with blue dye) R-T)geographic ulcerative lesions U)small intestine.

Comment:
This histologic fetures are not fully supportive of inflammatory bowel disease. Further clinicopathologic correlation is suggested.
#T-59420_2 #M-38000_2 0 1105 000000
#T-C4560_2 #M-09183_2 0 1105 000000
#T-59200_2 #M-09183_2 0 1105 000000

已電子簽章

敬告：本單僅供醫師參考使用，不具任何申請效力！！
請病患妥為保管，若不慎遺失需自負個人資料外洩責任！
